# Case Report: Targeting of individual somatic tumor mutations by multipeptide vaccination tailored for HLA class I and II presentation induces strong CD4 and CD8 T-cell responses in a patient with metastatic castration sensitive prostate cancer

**DOI:** 10.3389/fimmu.2023.1271449

**Published:** 2023-10-18

**Authors:** Henning Zelba, Armin Rabsteyn, Oliver Bartsch, Christina Kyzirakos, Simone Kayser, Marcel Seibold, Johannes Harter, Pauline Latzer, Dirk Hadaschik, Florian Battke, Alexander Golf, Matthew B. Rettig, Saskia Biskup

**Affiliations:** ^1^ Zentrum für Humangenetik, Tuebingen, Germany; ^2^ CeGaT GmbH, Tuebingen, Germany; ^3^ Cecava GmbH, Tuebingen, Germany; ^4^ MVZ Zentrum für ambulante Onkologie GmbH, Tuebingen, Germany; ^5^ Departments of Medicine and Urology, University of California, Los Angeles, Los Angeles, CA, United States; ^6^ Department of Medicine, VA Greater Los Angeles Healthcare System, Los Angeles, CA, United States

**Keywords:** prostate cancer, neoantigen, peptide, vaccination, APC

## Abstract

Localized prostate cancer is curable, but metastatic castration sensitive prostate cancer has a low 5-year survival rate, while broad treatment options are lacking. Here we present an mCSPC patient under remission receiving individualized neoantigen-derived peptide vaccination as recurrence prophylaxis in the setting of an individual treatment attempt. The patient was initially analyzed for somatic tumor mutations and then consecutively treated with two different peptide vaccines over a period of 33 months. The first vaccine contained predicted HLA class I binding peptides only whereas the second vaccine contained both predicted HLA class I and II binding peptides. Intracellular cytokine staining after 12 day in-vitro expansion measuring four T-cell activation markers (IFNg, TNF-α, IL-2, CD154) was used to determine vaccine-induced T-cell responses. While the first vaccine induced only one robust CD4+ T-cell response after 21 vaccinations, co-vaccination of HLA class I and II peptides induced multiple strong and durable CD4+ and CD8+ T-cell responses already after sixth vaccinations. The vaccine-induced immune responses were robust and polyfunctional. PSA remained undetectable for 51 months. The results presented here implicate that neoantigen-targeting vaccines might be considered for those cancer subtypes where therapeutic options are limited. Furthermore, our findings suggest that both HLA class I and II restricted peptides should be considered for future peptide vaccination trials.

## 1 Introduction

Prostate cancer, a malignancy that arises from the epithelial cells of the prostate gland, is the most common cancer among men and the second leading cause of cancer death in men (1). Localized prostate cancer is curable by surgery or radiotherapy as localized disease has a 5-year survival rate of almost 100%. However, metastatic castration sensitive prostate cancer (mCSPC) has a 5-year survival rate of only 29.8% and is generally considered to be incurable, clearly indicating that there is a clinical need for advanced treatment options (2). Current applied treatments for mCSPC include mainly androgen-deprivation therapy (ADT) to prevent prostate cancer cells from growth-stimulating androgen uptake (3).

Sipuleucel-T, a cancer vaccine activating the anti-PAP (prostatic acid phosphatase) immune response, was approved in 2010 (4). Other immunotherapeutic approaches, especially immune-checkpoint inhibitors like anti-PD-1 monoclonal antibodies (e.g. pembrolizumab), that are effective in many other cancer entities, showed only limited clinical activity in prostate cancer (5). Prostate cancer is traditionally considered as an immunologically “cold” tumor with low tumor mutational burden, limited T-cell infiltration, and an immunosuppressive tumor microenvironment (TME) (6, 7). However, the low number of shared immunogenic target mutations combined with strikingly decreased priming and/or (re)activation of neoantigen-specific T-cells could be overcome by vaccine strategies, especially in a personalized setting. Here we present a mCSPC patient under remission receiving individualized neoantigen-derived peptide vaccination as recurrence prophylaxis in the setting of an individual treatment attempt.

## 2 Case description

### 2.1 Patient

The patient is a 63-year-old caucasian male with recurrent metastatic castration sensitive prostate cancer. The patient was originally diagnosed in 2014 at the age of 54 when a palpable prostate nodule was detected (PSA 1.2). Prostate needle biopsy demonstrated Gleason 4 + 5 in four cores; staging scans including a bone scan and CT scan of the abdomen and pelvis did not reveal evidence of metastatic disease. The patient underwent a robotic laparoscopic radical prostatectomy with pelvic lymph node dissection in 2014 (staging: pT3aN0).

Subsequently the patient had a biochemical recurrence which prompted salvage radiation therapy in March, 2015 (03/2015) when the PSA level was 0.12. PSA further increased to 0.55, and the patient received sipuleucel-T in 05/2016. PSA increased to 1.0 in 11/2016, and a PSMA PET-CT revealed a PSMA avid right pelvic sidewall lymph node for which the patient underwent salvage right pelvic lymph node resection in 12/2016 (tumor sample I) and adjuvant whole pelvic node intensity modulated radiation. The PSA then reached a nadir of 0.03 in 08/2017, before starting to rise again to a peak of 13.4 in 05/2018. A PSMA PET-CT in 05/2018 showed two new PSMA avid right common iliac pelvic nodes, two cardiophrenic lymph nodes, and a right upper lobe ground glass opacity. Surgical resection of the cardiophrenic lymph nodes and right upper lobe lesion in 06/2018 (tumor sample II) resulted in pathologically confirmed prostate cancer. Stereotactic body radiation therapy to the common iliac nodes was performed from July to August of 2018. Germline sequencing did not identify any pathogenic variants, while evaluation of somatic variants of potential clinical relevance of tumor samples I and II by somatic tumor exome- and transcriptome analysis demonstrated prostate-common genomic driver mutations like *TP53* p.V157F and *APC* p.S1411N variants (8) as well as an *SLC45A3-BRAF* fusion.

**Figure 1:**
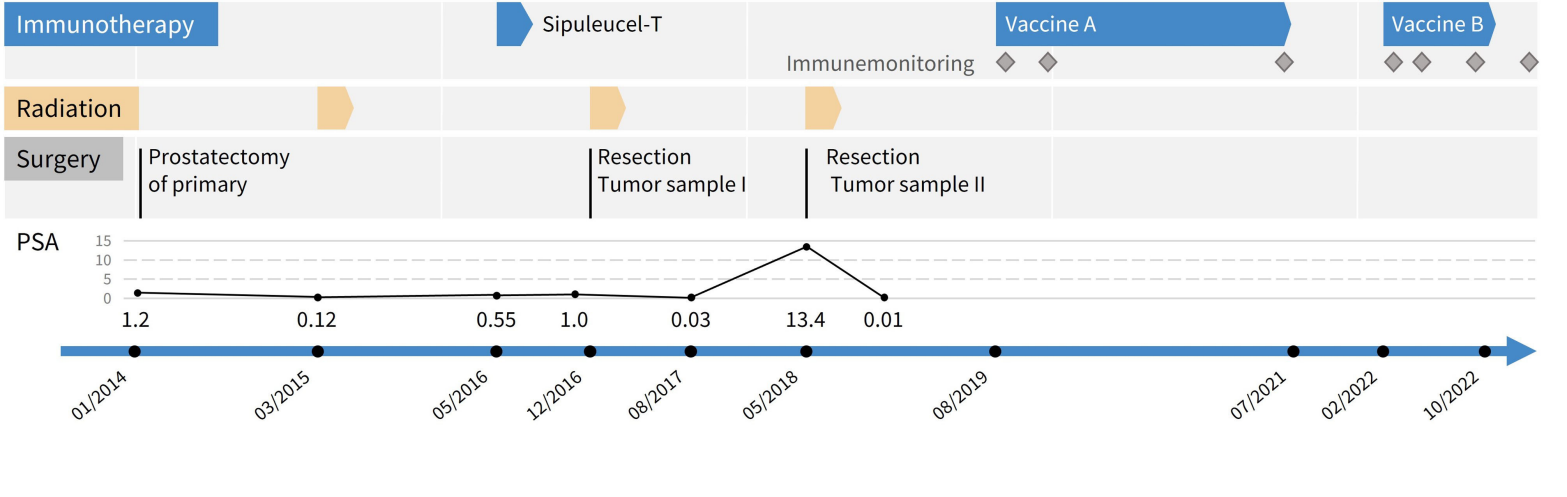
Timeline of clinical treatments from first diagnosis until end of peptide vaccinations. Grey boxes represent surgical interventions, yellow arrows episodes of radiation therapy, blue arrows immunotherapeutic approaches. Grey diamonds indicate immune monitoring time points.

At that time, leuprolide along with abiraterone and prednisone were initiated for mCSPC. The PSA rapidly declined to undetectable levels (<0.01 ng/ml) from 11/2018 until 02/2023. Since then, PSA has remained undetectable (**Figure 1**). Digital droplet PCR for the somatic *TP53* p.V157F and *APC* p.S1411N variants in cell-free DNA from whole blood remained negative.

### 2.2 Neoantigen-derived multipeptide vaccine

Vaccine design, formulation, and administration was performed as previously described (9). Briefly, somatic tumor variants were determined by exome analysis of DNA derived from tumor sample II (FFPE) and peripheral blood as normal tissue. Total RNA isolated from tumor was used for expression analysis of somatic tumor variants. HLA class I typing was performed and used for HLA class I epitope prediction of putative HLA class I binding peptides containing somatic variants. Peptides were selected based on binding prediction scores, allele frequencies, and RNA expression levels in the tumor sample. Putative HLA class II binding peptides were designed to span relevant tumor variants.

Peptides were synthesized and formulated into vaccines. For each vaccination, 0.5 ml multipeptide solution (0.8 mg/mL per peptide) was injected intracutaneously into the lower abdomen followed by subcutaneous injection of 83 µg sargramostim and superficial application of imiquimod. Formulation and administration were performed at the Zentrum für Humangenetik Tübingen, Germany.

### 2.3 Immune monitoring of vaccine-induced T-cell responses

Detection of neoantigen-specific T-cells was performed as previously described (9). Briefly, blood mononuclear cells (PBMC) including T cells were isolated by Ficoll Hypaque and cryopreserved for later use. Preserved PBMC were thawed and cells were cultured overnight to recover, stimulated with patient-individual mutated peptides and cultured 12 days in the presence of IL-2 and IL-7. For analysis, cells were briefly restimulated with peptides or incubated with DMSO (unstimulated negative control) or CytoStim™ (as unspecific positive control). Activated cells were measured after intracellular cytokine staining by flow cytometry. A detailed gating strategy can be found in **Supplementary Materials** (**Supplementary Figure 1**).

Peptide-specific responses were evaluated using the stimulation index (SI). The stimulation index is the calculated ratio of polyfunctional activated CD4+ or CD8+ T cells (positive for at least two markers of IFN-γ, TNF-α, IL-2 and/or CD154) in the peptide-stimulated sample to the negative control sample (DMSO). Neoantigen-specific T-cells are defined as being present for SI ≥2. For further details, see **Supplementary Materials**.

## 3 Results

### 3.1 Vaccine A

The first peptide prediction focused on HLA class I peptides only and was based on the tumor sample from 2018 (tumor sample II). This selection comprised 13 peptides (**Supplementary Table 1**). All respective targeted somatic variants were also present in tumor sample I from 2016. Altogether, the patient received 21 vaccinations from 08/2019 until 07/2021 (**Figure 1**).

**Figure 2:**
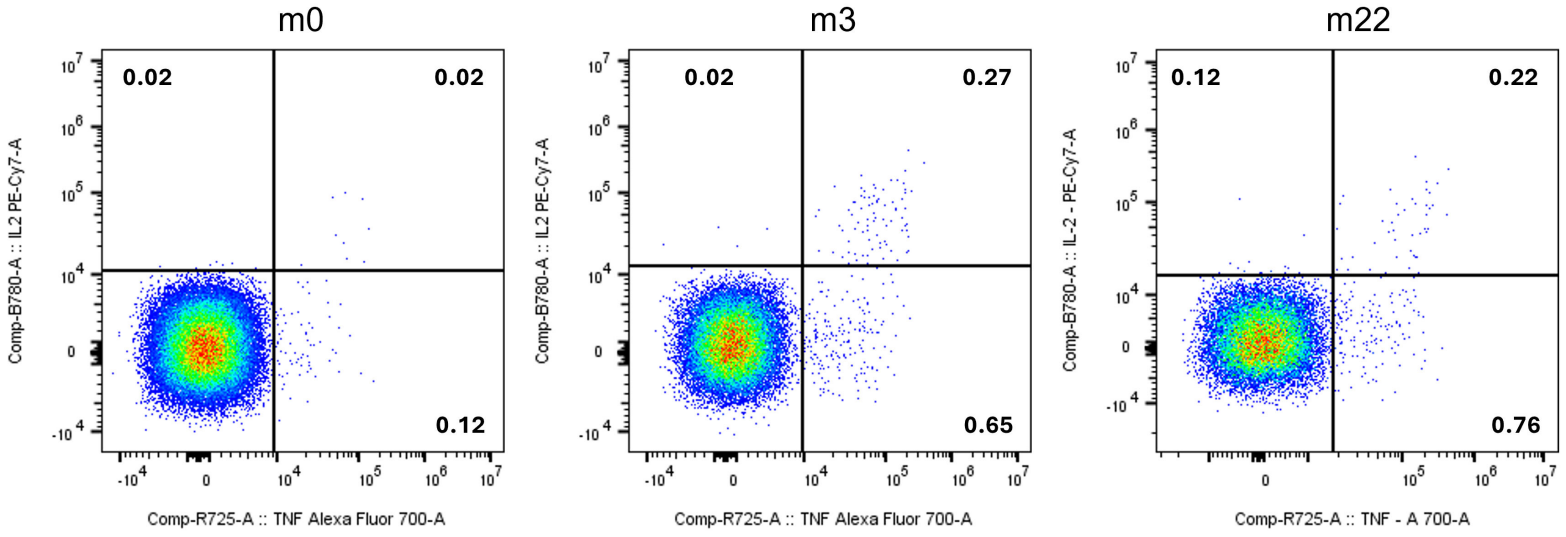
Vaccine-induced CD4+ T-cells specific for peptide 1 (VQSEPCNGMV; APC : NM_000038.6:c.4232G>A:p.S1411N) before (m0), three months (m3) and 22 months (m22) after the first injection of Vaccine A. T-cells are polyfunctional (y-axis: IL-2; x-axis: TNF-α). Numbers indicate frequency within all CD4+ T-cells.

Immune monitoring was performed before therapy and three as well as twenty-two months after the first injection. We detected a CD4+ T-cell response against peptide 1 (VQSEPCNGMV; APC p.S1411N) at three and 22 months (m3 and m22, respectively) after the first vaccination, but not before therapy (m0; **Figure 2**).

### 3.2 Vaccine B

Given that vaccine A induced only one immune response, a new vaccination cocktail was designed based on the same tumor sequencing analysis, but with updated HLA class I epitope prediction algorithms (see **Supplementary Information**). Additionally, putative HLA class II binding peptides were designed to cover variants with high allele frequencies and high RNA expression levels. The second selection included 20 peptides (9 HLA class I peptides, 11 HLA class II peptides; only peptide 1 from the previous peptide prediction was also included in vaccine B). The patient started with Vaccine B in 02/2022 and received 12 vaccinations until 12/2022.

Immune monitoring performed before the 1st application of vaccine B revealed a pre-existing CD4+ T-cell response against a pool of three HLA class II peptides (peptides 33, 34, 36; **Table 1**). Interestingly, vaccine A had included two HLA class I peptides targeting the same mutations (peptides 5 and 9).

**Table 1:** Summarized Immune monitoring results of Vaccine B.

Peptide No.	AA sequence	Gene	HLA	CD4				CD8			
1	VQSEPCNGMV	APC : NM_000038.6:c.4232G>A:p.S1411N	A*02:06	0,9	29,8	11,7	8,9	0,7	2,5	1,2	1
17	FVRGLQREL	CGN : NM_020770.3:c.1657C>T:p.L553F	C*12:03				1,1				1,1
18	TSPSYSPTY	POLR2A:NM_000937.5:c.5045C>A:p.S1682Y	C*12:03								
20	RVDHVMGSV	NEIL3:NM_018248.3:c.932A>G:p.D311G	A*02:06, C*12:03								
21	ALILTPTRV	DDX59:NM_001031725.6:c.845_846delAG:p.E282Vfs*10	A*02:06				1,1				0,9
23	TTPTGTQAAYTRPTVSP	EMSY : NM_020193.4:c.1525A>G:p.T509A	Class II	1,2	22,8	3,3	32,3	1,0	0,9	1,0	1,5
25	LQQIFESQHMKFSEIPQ	SMARCD1:NM_003076.5:c.1052G>A:p.R351H	Class II			16,8	2,4			1,0	3,6
26	PPPKVVDVSSHASQSAR	ATN1:NM_001940.4:c.2269C>T:p.P757S	Class II	1,0	129,1	22,2	10,3	0,8	0,9	1,5	1,9
30	ALILTPTRVSHSDRETS	DDX59:NM_001031725.6:c.845_846delAG:p.E282Vfs*10	Class II				101,0				2,7
31	KVHEEIERAIGANRAPS	CYP2U1:NM_183075.3:c.1154T>C:p.V385A	Class II				62,3				1,9
14	TRFRAMAIY	TP53:NM_000546.6:c.469G>T:p.V157F	C*12:03	1,0	1,1	1,0	1,0	0,8	1,2	2,4	0,8
15	TQAAYTRPTV	EMSY : NM_020193.4:c.1525A>G:p.T509A	A*02:06	1,1	1,4		1,0	0,7	29,2		1,1
16	TTWNILPSV	MT-CO1:ENST00000361624:m.7264T>C:p.S454P	A*02:06, C*12:03				0,8				1,3
19	VHEEIERAI	CYP2U1:NM_183075.3:c.1154T>C:p.V385A	B*38:01				1,0				1,1
22	STPPPGTRFRAMAIYKQ	TP53:NM_000546.6:c.469G>T:p.V157F	Class II	1,6	1,6	16,0	7,2	0,8	1,6	0,8	1,9
24	SSVQSEPCNGMVSGIIS	APC : NM_000038.6:c.4232G>A:p.S1411N	Class II	1,2	85,4		31,1	1,0	0,9		1,1
32	SKGLLPNNLEESGICHK	XPR1:NM_004736.4:c.1301C>T:p.S434L	Class II				6,1				1,2
33	EEAEESTPTQKRKGRQS	TSHZ3:NM_020856.4:c.2662G>A:p.A888T	Class II	3,6	27,4		1,0	0,7	1,4		1,1
34	SSRVDHVMGSVARKSEE	NEIL3:NM_018248.3:c.932A>G:p.D311G	Class II			12,9	31,5			1,2	2,0
36	PGPSDPGPDVNRTESPM	PRRT3:NM_207351.5:c.1130C>A:p.A377D	Class II			1,3				0,5	
				M0	M3	M8	M13	M0	M3	M8	M13

AA, amino acid; HLA, Predicted HLA I restriction. Numbers indicate the Stimulation Index (SI): ratio of polyfunctional activated CD4+ or CD8+ T-cells (positive for at least two activation markers of CD154, IFN-γ, TNF-α and/or IL-2) in the peptide-stimulated sample compared to the unstimulated control. Blue boxes indicate presence of neoantigen-specific T-cells. Neoantigen-specific T-cells are defined as being present for SI >2. Due to low cell numbers, some peptides were analysed in pools (e.g. peptide no. 1, 17, 18, 20, 21). Mx: months after first vaccination of Vaccine B. *: this peptide was included in Vaccine A as well.

Three months after vaccination start (M3; after 6 vaccinations), CD4+ as well as CD8+ T-cell responses against 6 of 8 peptide pools were detectable. The observed immune responses were strong (between 0.5% and 27.5% of CD4+ and CD8+ T-cells respectively) and polyfunctional (at least two of four markers were detected).

Eight months after vaccination start (M8; after 11 vaccinations), strong and polyfunctional CD4+ and CD8+ T-cell responses against 7 of 8 peptide pools were detected. Most M3 responses were confirmed, and some previously pooled analyses could be ascribed to single peptides (peptides 23, 25 and 34).

**Figure 3:**
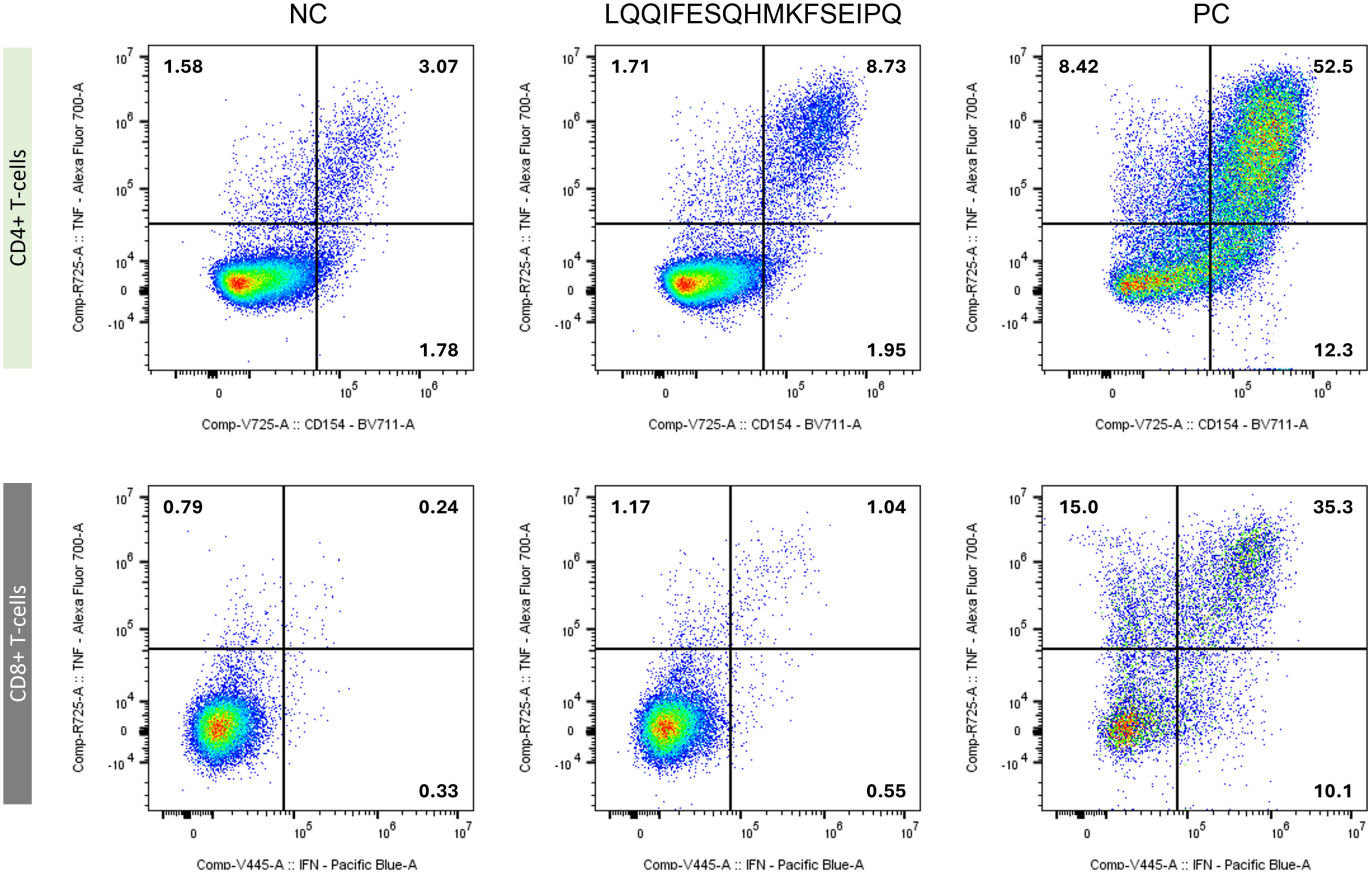
Immune monitoring result for peptide 25 (LQQIFESQHMKFSEIPQ; SMARCD1:NM_003076.5:c.1052G>A:p.R351H) from M13. Vaccine-induced mutated SMARCD1-specific CD4+ T-cells (upper row; y-axis: TNF-α; x-axis: CD154) and CD8+ T-cells (lower row; (y-axis: TNF-α; x-axis: IFN-γ) are shown. T-cells were either mock restimulated (negative control, NC: (left)), peptide-restimulated cells (middle), or Cytostim-stimulated sample (positive control, PC: (right)). Numbers indicate frequency within all CD4+ or CD8+ T-cells, respectively.

Thirteen months after vaccination start (M13), strong and polyfunctional CD4+ as well as CD8+ T-cell responses against 10 of 17 peptide pools were detectable (for example against peptide 25; see **Figure 3**). Again, previously pooled analyses could be ascribed to single peptides; including peptides 1 and 24, derived from the APC p.S1411N variant (**Supplementary Figure 2**).

## 4 Discussion

Immunotherapy was established as the fourth pillar of cancer treatment in recent years. However, most cancer patients do still not benefit from approved immunotherapeutic approaches like immune-checkpoint inhibitors. Thus, personalized vaccination against tumor-specific (neo)antigens is currently an intensively investigated research area. Here we present a recurrent metastatic castration sensitive prostate cancer patient undergoing personalized neoantigen-derived peptide vaccination as recurrence prophylaxis. Peptide-based vaccines comprising synthetic peptides have the advantage of being well-defined and relatively cost effective to manufacture while ensuring safety and feasibility.

In order to identify sufficient target mutations, most vaccine-based treatments were so far mainly performed in TMB high tumors like melanoma (10, 11). Prostate cancer generally has much lower TMB (12), however we were still able to identify an adequate number of potential neoantigens in the presented case. Although there are many treatments approved for advanced prostate cancer, neoantigen-targeting vaccines might be considered for patients with limited therapeutic options and high recurrence rates.

When we initially designed the vaccine in 2019, Evidence for (therapeutic) efficacy of CD4+ T-cell-mediated targeting of HLA class II presented peptides was low (13, 14). Hence, the first vaccine included HLA class I peptides only. During therapy, the patient established a moderate vaccine-induced CD4+ T-cell response against one neoantigen. As we did not induce any additional responses after 20 vaccinations, we decided to re-evaluate existing sequencing datasets and include putative neoepitopes presented on HLA class II.

Strikingly, already after the 6^th^ injection of this new vaccine, immune monitoring revealed strong CD4+ and/or CD8+ T-cell responses against at least 9 peptides, including a strong CD4 response against the *APC* p.S1411N variant. During the course of treatment, vaccine-induced responses remained robust, durable, and polyfunctional. We previously observed similar results in a urothelial carcinoma patient (15) as well as four breast cancer patients (9).

We noticed no serious adverse events (SAEs) during vaccination. Minor temporal local skin reactions at vaccination sites such as redness, itching, and swelling resolved without interventions. This is in consensus with previously reported results using peptide-based vaccines combined with sargramostim in larger trials (16, 17).

We provide further evidence that it is technically feasible to produce a fully individualized neoantigen-derived peptide vaccine in an adjuvant setting. We were able to induce/enhance T-cell responses against a variety of target neoantigens without any additional treatment, such as immune-checkpoint inhibition. Although the follow-up time might be too short to encompass a possible recurrence, the patient has remained in remission and PSA has been undetectable since November 2018. Digital droplet PCR for the somatic *TP53* and *APC* variants in cell-free DNA remained negative.

In line with these findings, Karbach et al. have demonstrated in a recently published case report the ability of tumor-specific T-cells to keep a prostate cancer patient in durable complete remission (18).

Strikingly, to the best of our knowledge, this is the first report of a somatic APC mutated prostate cancer responding to any form of immunotherapy. APC mutations occur in about 2% of all PCAs (19). Considering current case numbers, this means that there are more than 20,000 PCA patients per year in the US and Europe with a so far unmet need for optional therapies.

There are numerous emerging research questions that may warrant further investigations in larger cohorts, such as optimization of prediction algorithms and foremost the optimal ratio/amount of co-vaccinated HLA class I and II binding peptides to further improve (personalized) peptide vaccine approaches.

## 5 Patient perspective

The presented case implies a promising example of a mCSPC patient who remained in remission undergoing personalized neoantigen vaccination. We have shown that it is technically feasible to produce and apply a potent neoantigen-derived peptide vaccine. Production and formulation of each vaccine was finished about 10 weeks after initiation. The application was safe. Robust and polyfunctional T-cell responses were induced or enhanced without any additional treatment such as immune-checkpoint inhibition. If more encouraging data can be gathered, neoantigen-derived peptide vaccines have the potential to be used not only in the adjuvant setting, but also as an interventional treatment in newly diagnosed patients.

## Data availability statement

The raw data supporting the conclusions of this article will be made available by the authors, without undue reservation.

## Ethics statement

Ethical approval was not required for the studies involving humans because individual treatment attempts are not dependent on ethics approval in Germany. The study was conducted in accordance with the local legislation and institutional requirements. The participant provided written informed consent to participate in this study. Written informed consent was obtained from the individual for the publication of any potentially identifiable images or data included in this article. Written informed consent was obtained from the participant/patient(s) for the publication of this case report.

## Author contributions

HZ: Investigation, Methodology, Project administration, Supervision, Visualization, Writing – original draft, Writing – review & editing. AR: Formal Analysis, Investigation, Methodology, Writing – review & editing. OB: Formal Analysis, Investigation, Methodology, Writing – review & editing. CK: Conceptualization, Formal Analysis, Investigation, Methodology, Writing – review & editing. SK: Conceptualization, Formal Analysis, Investigation, Methodology, Writing – review & editing. MS: Conceptualization, Formal Analysis, Investigation, Methodology, Writing – review & editing. JH: Formal Analysis, Investigation, Writing – review & editing. PL: Formal Analysis, Investigation, Writing – review & editing. DH: Conceptualization, Formal Analysis, Methodology, Writing – review & editing. FB: Formal Analysis, Methodology, Software, Visualization, Writing – review & editing. AG: Formal Analysis, Investigation, Supervision, Writing – review & editing. MR: Formal Analysis, Investigation, Supervision, Writing – review & editing. SB: Investigation, Project administration, Resources, Supervision, Writing – original draft, Writing – review & editing.
