# Supplementary material for: Case Report: Targeting of individual somatic tumor mutations by multipeptide vaccination tailored for HLA class I and II presentation induces strong CD4 and CD8 T-cell responses in a patient with metastatic castration sensitive prostate cancer
Source: Front Immunol. 2023 Oct 18;14:1271449. doi: 10.3389/fimmu.2023.1271449 (PMC10619716; doi:10.3389/fimmu.2023.1271449)
Supplement: Supplementary file 1 [file DataSheet_1.docx]

**Supplementary Material for:**

Targeting of individual somatic tumor mutations by multipeptide vaccination tailored for HLA class I and II presentation induces strong CD4 and CD8 T-cell responses in a patient with metastatic castration sensitive prostate cancer

**Author List and Affiliations:**

Henning Zelba^1^, Armin Rabsteyn^1^, Oliver Bartsch^1^, Christina Kyzirakos^1^, Simone Kayser^1^, Marcel Seibold^1^, Johannes Harter^2^, Pauline Latzer^1^, Dirk Hadaschik^3^, Florian Battke^2^, Alexander Golf^4^, Matthew B Rettig^5,6^, Saskia Biskup^1,2,4^

1. Zentrum für Humangenetik, Tuebingen, Germany
2. CeGaT GmbH, Tuebingen, Germany
3. Cecava GmbH, Tuebingen, Germany
4. MVZ Zentrum für ambulante Onkologie GmbH
5. Departments of Medicine and Urology, University of California, Los Angeles, CA, USA
6. Department of Medicine, VA Greater Los Angeles Healthcare System

**Corresponding author:**

Henning Zelba

Zentrum für Humangenetik

Paul-Ehrlich-Str. 23

72076 Tübingen, Germany

E-Mail: henning.zelba@humangenetik-tuebingen.de


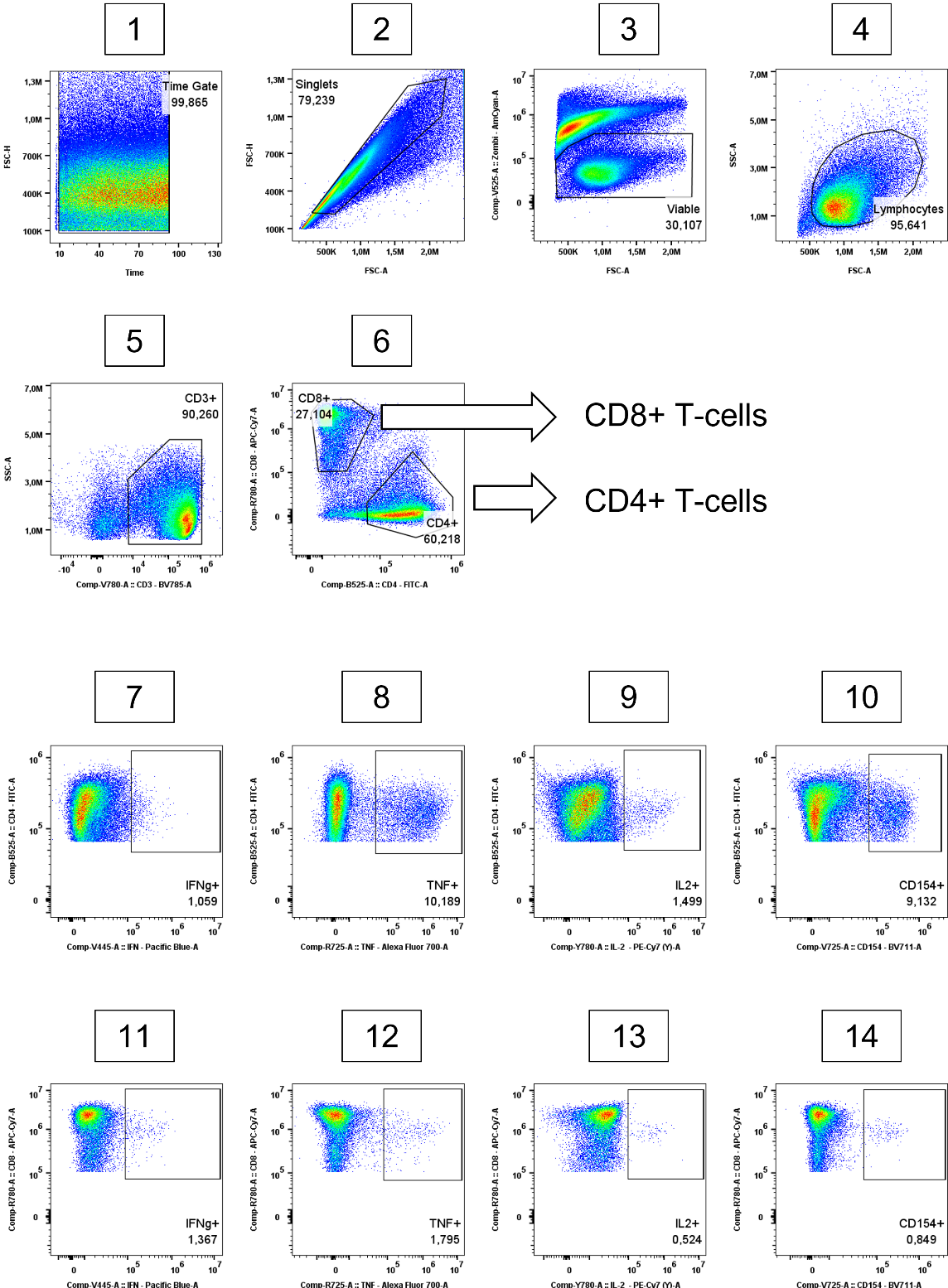
 **Supplementary Figure 1:**

Gating strategy:

We included only cells that were constantly measured over time (1; Forward-scatter (FSC)-H versus Time). Herein, single (2; FSC-A versus FSC-H), viable (3; Zombie Aqua-negative cells), lymphocytes (4; FSC-A versus Side-scatter (SSC)-A) and CD3+ T cells (5) were selected. CD3+ T cells were further discriminated in CD4+ or CD8+ T cells (6). Within both CD4+ (7-10) and CD8+ T cells (11-14), we determined the production/expression of the functional markers IFN-γ (7, 11), TNF-α (8, 12), IL-2 (9, 13) and CD154 (10, 14). Boolean gating strategy was used to determine T-cells that produce/express more than one functional markers.

| **Peptide No.** | **AA sequence** | **Gene** | **HLA** | **CD4** | | | **CD8** | | |
| --- | --- | --- | --- | --- | --- | --- | --- | --- | --- |
| 1 | VQSEPCNGMV | APC:NM_000038:c.G4232A:p.S1411N | HLA-A*02:06 | 1,0 | 3,5 | 3,1 | 1,0 | 0,5 | 1,7 |
| 2 | STPPPGTRF | TP53:NM_000546:c.G469T:p.V157F | HLA-C*12:03 | 1,2 | 1,2 | 1,7 | 2,0 | 1,0 | 3,2 |
| 3 | SQHMKFSEI | SMARCD1:NM_003076:c.G1052A:p.R351H | HLA-A*02:06 HLA-C*12:03 | 1,9 | 0,9 |  | 1,0 | 1,3 |  |
| 4 | RQKMSAFV | CGN:NM_020770:c.C1657T:p.L553F | HLA-A*02:06 | 0,8 | 0,9 |  | 0,8 | 0,8 |  |
| 5 | DVNRTESPM | PRRT3:NM_207351:c.C1130A:p.A377D | HLA-A*26:01 | 1,6 | 0,7 |  | 1,0 | 1,3 |  |
| 6 | RAIGANRAPSL | CYP2U1:NM_183075:c.T1154C:p.V385A | HLA-C*12:03 | 1,0 | 1,1 |  | 1,6 | 1,2 |  |
| 7 | KVVDVSSHA | ATN1:NM_001940:c.C2269T:p.P757S | HLA-A*02:06 | 0,9 | 0,6 |  | 0,5 | 0,4 |  |
| 8 | YSPTYPSYSPT | POLR2A:NM_000937:c.C5045A:p.S1682Y | HLA-A*02:06 | 0,9 | 1,2 |  | 1,2 | 0,6 |  |
| 9 | RVDHVMGSV | NEIL3:NM_018248:c.A932G:p.D311G | HLA-C*12:03 | 1,2 | 1,0 |  | 1,1 | 0,5 |  |
| 10 | LLPNNLEESGI | XPR1:NM_004736:c.C1301T:p.S434L | HLA-A*02:06 | 1,4 | 0,8 |  | 1,6 | 1,4 |  |
| 11 | CQGEREKAATL | TUFT1:NM_020127:c.C806G:p.A269G | HLA-A*02:06, HLA-C*08:03 |  | 0,9 |  |  | 1,0 |  |
| 12 | SSLLKHTM | OSBPL8:NM_020841:c.G815A:p.R272H | HLA-A*02:06 | 1,0 | 1,0 |  | 0,8 | 0,6 |  |
| 13 | ALRQGIGL | SPECC1L:NM_015330:c.G2475A:p.M825I | HLA-A*02:06 | 1,1 | 0,6 |  | 0,8 | 0,8 |  |
|  |  |  |  | m0 | m3 | m22 | m0 | m3 | m22 |

Supplementary Table 1: Summarized Immune monitoring results of Vaccine A

AA: amino acid. HLA: HLA which was predicted to bind the peptide. Numbers indicate the Stimulation Index (SI): ratio of polyfunctional activated CD4+ or CD8+ T cells (positive for at least two activation markers of CD154, IFN-γ, TNF and/or IL-2) in the peptide-stimulated sample compared to the unstimulated control. Blue boxes indicate presence of neoantigen-specific T-cells. Neoantigen-specific T-cells are defined as being present for SI ≥2. Due to low cells numbers, some peptides were analysed in pools (e.g. peptide no. 10 and 11). mx: months after first vaccination of Vaccine B. Grey boxes: this peptide was included in Vaccine B as well.

**Supplementary Figure 2:**


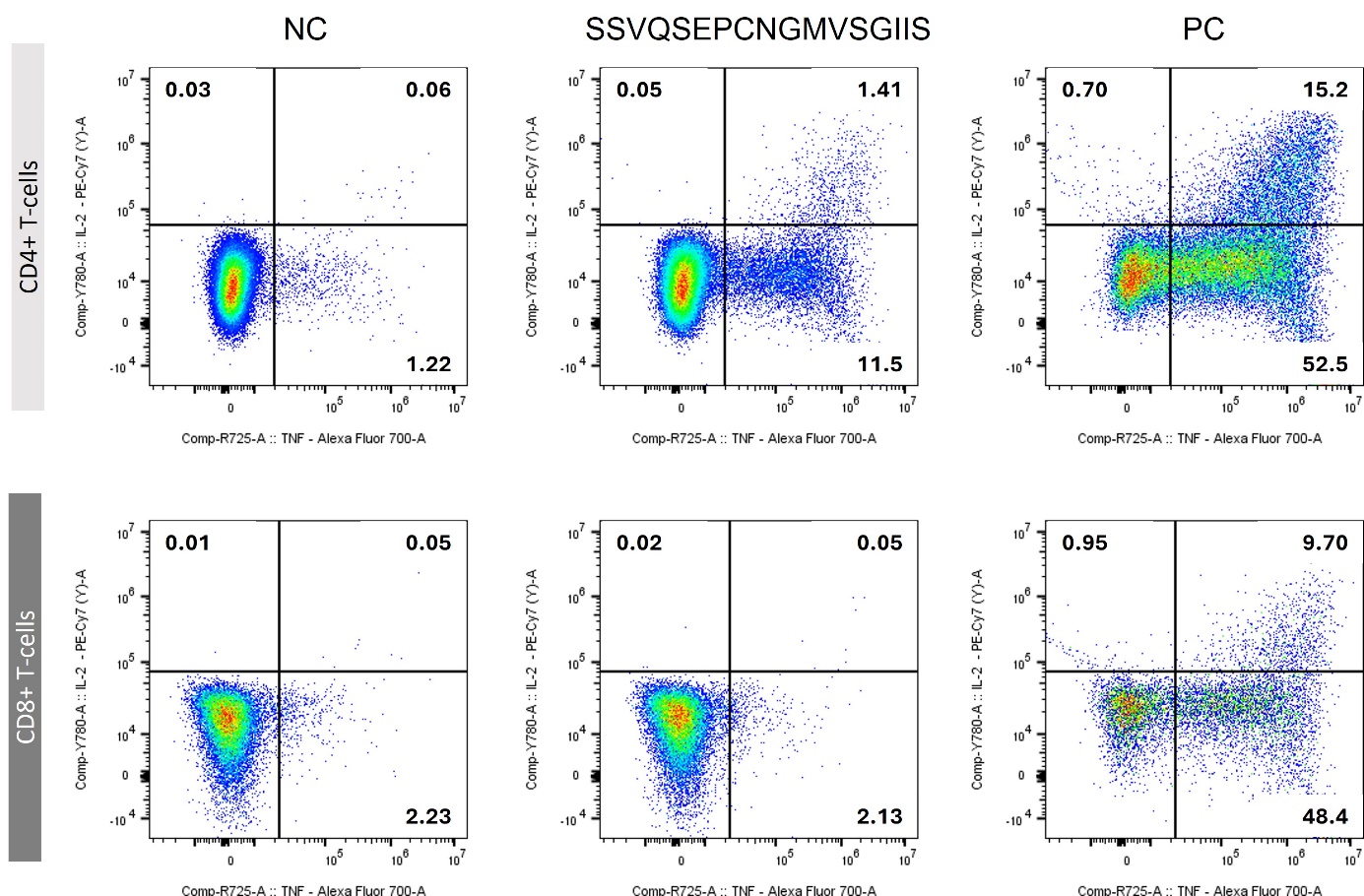


Immune monitoring result for peptide 24 (APC:NM_000038.6:c.4232G>A:p.S1411N) from M13. Vaccine-induced peptide-specific CD4+ T-cells (upper row) and are polyfunctional (x-axis: TNF; y-axis: IL-2). Neoantigen-specific CD8+ T-cells (lower row) were not detected. Mock restimulated sample (negative control, NC: (left)), peptide-restimulated cells (middle), and Cytostim-stimulated sample (positive control, PC: (right)) are shown. Numbers indicate frequency within all CD4+ or CD8+ T-cells, respectively.

**Supplementary Methods:**

DNA sequencing: The coding and flanking intronic regions were enriched using in solution hybridization technology and were sequenced using the Illumina HiSeq/NovaSeq system (Illumina).

Computational analysis: Illumina bcl2fastq2 was used to demultiplex sequencing reads. Adapter removal was performed with Skewer. The trimmed reads were mapped to the human reference genome (hg19) using the Burrows Wheeler Aligner. Reads mapping to more than one location with identical mapping score were discarded. Read duplicates that likely result from PCR amplification were removed. The remaining high-quality sequences were used to determine sequence variants (single nucleotide changes and small insertions/deletions). The variants were annotated based on several internal as well as external databases.

Genetic data evaluation: Only variants (SNVs/small indels) with a novel allele frequency (NAF) of ≥ 5% in the tumor sample within the coding regions and their adjacent intronic regions (-/+ 8 base pairs) were evaluated. Variants frequently observed in-house (> 5%) are excluded. The sensitivity of the test is dependent on the tumor content of the analyzed material, the sample quality, and the sequencing depth.

Variants are named according to the HGVS recommendations. The sample fulfilled our quality criteria upon arrival and during/after each processing step in the laboratory. The procedure described above was developed and validated in-house (Laboratory developed test; LDT). A minimal tumor content of 20 % was taken as threshold.

RNA sequencing: Library preparation was performed using the TruSeq Total RNA (RiboZero rRNA removal Kit) or- SMARTer Stranded Total RNA Library Kit and subsequently analyzed using high-throughput sequencing on the HiSeq/NovaSeq system (Illumina). Mapping of sequencing reads to the hg19 reference genome was performed with STAR (Version 2.5.2b). Gene expression analysis (counting of aligned reads per gene, calculation of normalized read counts and calculation of FPKM values) was done with DESeq2 (Love et al., 2014, PMID: 25516281) in R (R Core Team 2015).

Prediction of neoepitopes: Somatic missense variants only present in the tumor but absent in the normal tissue of the patient were identified by whole exome sequencing analysis. Identified somatic missense variants (SNVs) were sent to the Center for Bioinformatics Tübingen, Dept. of Computer Science, University Tübingen for epitope prediction and HLA typing (Szolek et al., 2014, PMID: 25143287). HLA class I epitopes for cocktail A were predicted using SYFPEITHI, netMHC-3.0 and netMHCpan-2.4 (Rammensee et al., 1999, PMID: 10602881; Lundegaard et al., 2008, PMID: 18463140; Hoof et al., 2009, PMID: 19002680; vaccine A). HLA class I epitopes for cocktail B were predicted using SYFPEITHI, netMHC-4.0 and netMHCpan-3.0 (Rammensee et al., 1999, PMID: 10602881; Andreatta et al., 2016, PMID: 26515819; Nielsen et al., 2016, PMID:27029192; vaccine B). Peptides containing somatic variants that were classified as HLA class I binder by at least one prediction method were further evaluated. The respective thresholds for classification as binder are defined as <500 nM for netMHC and netMHCpan as well as >50% of maximal score for SYFPEITHI. Peptides resembling a wildtype sequence in the human proteome (based on UniProtKB/Swiss-Prot, human, 9/7/14) were excluded.

Selection of peptides: Peptides derived from genes not expressed in the patient’s tumor were excluded. For this purpose, expression data for the respective variant were analyzed using RNA sequencing data of the tumor sample. Putative HLA class I epitopes with a high HLA class I binding prediction score derived from variants with high allele frequencies in the tumor were selected. Peptides predicted to bind to different HLA class I molecules of the patient were prioritized. Peptides which are predicted to bind to several HLA molecules of the patient were further prioritized.

Putative HLA class II epitopes with a length of +/-17 amino acids were designed to contain variants with high allele frequencies in the tumor (vaccine B only). Peptides spanning variants in tumor driver genes were prioritized. Excluded were peptides with a high percentage of hydrophobic amino acids, with a high probability for gelation or dimerization to avoid solubility problems in the aqueous vaccine solution or problems during peptide synthesis.

The bioinformatically identified somatic variants corresponding to all selected peptides were manually reviewed in the sequencing data and filtered for false positives.

Immune monitoring:

Blood mononuclear cells (PBMC) including T cells were isolated by Ficoll Hypaque and cryopreserved for later use. Preserved PBMC were thawed and cells were cultured overnight to recover, stimulated with patient-individual mutated peptides and cultured 12days in the presence of low dose IL 2 and IL 7. This led to an amplification of specific T cell responses and to a higher sensitivity of the analysis. For analysis, cells were briefly restimulated with peptides or incubated with DMSO (unstimulated negative control) or CytoStim™ (as unspecific positive control) in presence of Golgi-Plug (BD biosciences) at a concentration of 1 µl/ml.

After restimulation, the final readout was an Intracellular Cytokine Staining (ICS). After cultivation, cells were washed twice followed by extracellular staining with fluorochrome-conjugated antibodies titrated to their optimal concentrations: CD3-BV785 (clone UCHT1; BioLegend), CD4-FITC (clone RPA-T4; BioLegend), CD8-APC/Cyanine (clone SK1; BioLegend), Zombi Aqua Dye (BioLegend).

After extracellular staining, cells were fixed and permeabilized (BD biosciences), followed by an intracellular staining with the following antibodies: IFN-BV421 (clone 4S.B3; BioLegend), TNF-AlexaFluor700 (clone MAb11; BioLegend), IL-2-PE/Cy7 (clone MQ1-17H12; BioLegend) and CD154 – BV711 (clone 24-31; BioLegend). Finally, cells were measured on a Novocyte 3005R cytometer (ACEA biosciences).

Evaluation of specific responses: Peptide-specific responses were evaluated using the stimulation index (SI). The stimulation index is the calculated ratio of polyfunctional activated CD4+ or CD8+ T cells (positive for at least two markers of IFN-γ, TNF, IL-2 and/or CD154) in the peptide-stimulated sample to the negative control sample (DMSO). Neoantigen-specific T-cells are defined as being present for SI ≥2. Additionally, a minimal frequency of 0.1% of reactive T cells positive for at least one activation marker including IFN-γ, TNF, Il-2 and/or CD154 must be reached among a minimum of 10 000 measured CD4+ or CD8+ events or a minimal frequency of 0.2% of reactive T cells positive for at least one activation marker among a minimum of 5 000 measured CD4+ or CD8+ events.
